# Improving SLAM‐Based Navigation in Flexible Ureteroscopy by Kidney Stone and Surgical Tool Segmentation

**DOI:** 10.1049/htl2.70038

**Published:** 2025-12-09

**Authors:** Laura Oliva‐Maza, Florian Steidle, Julian Klodmann, Klaus H. Strobl, Arkadiusz Miernik, Rudolph Triebel

**Affiliations:** ^1^ Institute of Robotics and Mechatronics German Aerospace Center (DLR) Wessling Germany; ^2^ Department of Urology, Faculty of Medicine University of Freiburg ‐ Medical Centre Freiburg Germany; ^3^ Karlsruhe Institute of Technology (KIT) Karlsruhe Germany

**Keywords:** image segmentation, kidney, medical image processing

## Abstract

Flexible ureteroscopy is a widely used surgical procedure for diagnosing and treating various urinary tract conditions, particularly kidney stones. Ensuring the complete extraction of all stones is crucial to prevent recurrence and the need for auxiliary interventions. Visual SLAM‐based navigation systems have been proposed to assist surgeons by simultaneously estimating the 3D structure of the kidney and tracking the ureteroscope's tip position. However, most existing solutions assume a completely static environment, which does not account for the intraoperative situation. In this study, we extend the work of Oliva Maza et al. by incorporating real‐time visual segmentation of kidney stones and surgical tools using either YOLOv7‐E6E and segment anything or YOLO11m‐seg. Our method discards pixels corresponding to instruments due to their inherent dynamic nature, while kidney stone pixels are incorporated into the SLAM framework but classified as potentially dynamic map points, allowing for their disappearance. This refinement enhances the robustness and the accuracy of ureteroscope position estimation for surgical navigation. To evaluate our approach, we recorded multiple datasets for both segmentation and ureteroscope pose estimation. Experimental results show an average improvement in ureteroscope pose estimation of 35.4% when using YOLOv7‐E6E with SAM, and 52.49% when using YOLO11m‐seg.

## Graphical abstract

Navigation in flexible ureteroscopy is challenging due to its dynamic environment. We propose to segment the possible dynamic objects (kidney stones and surgical tools) and use the segmentation mask in the SLAM system to improve its accuracy.

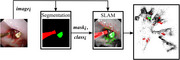

## 1 Introduction

Kidney stones are developed by 7%–13% of North Americans, 5%–9% of Europeans, and 1%–5% of Asians during their lifetime [1]. Depending on the size, location and composition of the stone, there are different treatment possibilities [2]. A common treatment is flexible ureteroscopy (fURS) where a flexible ureteroscope is introduced through the urinary tract to the kidney. Then the organ is explored and kidney stones can be removed using tools like baskets that are inserted through the working channel of the flexible ureteroscope [2]. When a stone is grasped, the tool and the stone are extracted through the working channel. Depending on the stone size, they can be either directly extracted or a prior step, where the stone is fragmented into smaller pieces using a laser must be performed. In order to avoid a subsequent intervention it is important, that all stones and stone fragments are found and extracted during the procedure.

The use of fURS is increasing due to the small number of contraindications [3], but it imposes significant challenges to the surgeon, including a steep learning curve, unergonomic positions, the necessity of two surgeons to work together in a confined space, and a limited field of view, yielding constrained spatial awareness due to the monocular endoscopic video [4].

The use of robotic systems helps to overcome some of these limitations [5, 6, 7], allowing a single surgeon to perform the procedure in a more ergonomic position. Additionally, computer vision methods that use visual simultaneous localization and mapping (vSLAM) can estimate a 3D map of the organ and track the pose of the flexible ureteroscope's tip within that map, enhancing visualization and navigation, and ultimately reducing the risk of missed stones.

However, vSLAM faces challenges such as rapid camera motion, lighting changes, reflections, lack of texture, a small field of view, and poor image quality [4]. Oliva Maza et al. [8], extend ORB‐SLAM3 to address these challenges and accurately estimate a 3D map of the kidney and the endoscope tip's pose. However, surgeons must still manually explore the entire kidney to ensure that no stones remain. A limitation here is the assumption of a static environment, which degrades performance when tools move or the kidney stones were already extracted.

To address this issue, detecting and segmenting kidney stones and tools in the endoscopic image can help mark stones on the 3D map, making them easier to locate and extract. Futhermore, the segmentation of tools informs the vSLAM system about dynamic areas, improving accuracy and functionality. Our contribution is the integration of segmentation masks of surgical tools and kidney stones into the SLAM system from Oliva Maza et al. [8]. The masks will be used to ignore the keypoints detected on surgical tools, improving its accuracy, and to mark stone locations on the 3D map, assisting the surgeon in navigating the kidney and in ensuring that all stones are removed.

## 2 State of the Art

In this section, we examine the primary approaches to simultaneous localization and mapping (SLAM) and segmentation, with an emphasis on methods focused on medical imaging.

### 2.1 Simultaneous Localization and Mapping

SLAM is a technique to estimate the 3D map of the environment that surrounds the camera and the pose of the camera in real time. Visual SLAM (vSLAM) uses only the information captured by passive cameras to estimate the map and the poses. ORB‐SLAM [9] is a state of the art keyframe‐based SLAM system that uses ORB (oriented FAST and rotated BRIEF) features [10], excelling in map reusability, loop closure, and relocalization. Whereas ORB‐SLAM [9] only considers monocular cameras, ORB‐SLAM2 [11] allows for stereo and RGB‐D cameras. Lastly, ORB‐SLAM3 [12] includes visual‐inertial SLAM and can handle multiple maps, to be eventually merged after loop closure. Furthermore, this system has been thoroughly researched for use in laparoscopy. Mahmoud et al. research [13, 14] focus on increasing the map density of ORB‐SLAM [9], enabling it to accurately track the endoscope in laparoscopic sequences. Lin et al. [15] extend ORB‐SLAM2[11] by deblurring the endoscopic images and substituting the ORB feature extractor by a blood vessel feature extractor being able to track more stable features. Oliva Maza et al. [8], extend ORB‐SLAM3 to work in the urinary tract by adding a preprocessing step to increase the contrast of the image, estimating a mask to avoid detecting features in non‐desired regions like reflections, and substituting ORB features by A‐KAZE (accelerated KAZE) features [16]. On the other hand, Fu et al. [17] use an electromagnetic sensor and integrate it on a SLAM system to track the tip of the endoscope and reconstruct a dense, metric 3D map of the kidney with an accuracy of 0.6 mm. They also provide the size of the stones by using adaptive threshold and watershed segmentation methods to distinguish the stone from the background and then use the 3D estimated pointcloud to compute its size.

In this paper, we focus on improving the work of Oliva Maza et al. [8] by integrating surgical tools and kidney stone segmentation. We base our approach on the extended version of ORB‐SLAM3 by Oliva Maza et al. [8] due to its real‐time capabilities, its proven robustness in urinary tract sequences and because it does not require additional sensors. The primary weakness of that approach is that a static environment was assumed, whereas the urinary tract and particularly the kidney feature dynamic objects such as tools and stones.

### 2.2 Segmentation of Surgical Tools, Solids, and Organs

The real‐time estimation of the size of kidney stones is critical information that assists surgeons in deciding whether further laser fragmentation is necessary or if the stone can be directly extracted using a basket. The process begins with computer vision techniques to detect, localize, and accurately segment the image region corresponding to the kidney stone. By integrating these segmentation masks into a SLAM system, the stone's location can be annotated within the 3D map. Additionally, the segmented image regions corresponding to surgical tools can be passed to the SLAM system as dynamic regions, such that they are not treated as static parts of the environment [18].

Convolutional neural networks (CNNs) are extensively utilized for object segmentation due to their great performance. However, the main difficulty when applying deep learning approaches to minimally‐invasive surgery (MIS) is the lack of large annotated datasets. U‐Net [19] is an encoder‐decoder architecture commonly used for medical image segmentation, as it can be trained effectively with a smaller number of images. Gupta et al. [20] and Li et al. [21] focus on training U‐Nets on expelled kidney stones with white or black background, without evaluating their performance or generalization within the internal organ environment. Segpromt [22] finetunes models by leveraging segmentation maps as prompts, which necessitates accurate training annotations to function effectively.

Several studies have focused on laparoscopic tool segmentation [23, 24, 25], but to our knowledge, there is a lack of works on the segmentation of ureteroscopic tools.

In this work, we compare two approaches for the segmentation of tools and kidney stones: the combination of the object detector YOLOv7‐E6E [26] with the segment anything model [27] (SAM, specifically MobileSAM [28]) (YOLO+SAM), and the YOLO11m‐seg model [29] (YOLO‐seg). We selected YOLO+SAM due to its robustness and the efficiency of its data labelling process, where accurate segmentation masks can be generated from bounding boxes. This is particularly advantageous for fine tuning YOLOv7‐E6E in our target domain. In contrast, YOLO‐seg is a single neural network capable of both object detection and segmentation, eliminating the need for a separate model. However, fine‐tuning YOLO11m‐seg requires mask‐level segmentation annotations, which constitute a considerably more complex labelling task.

## 3 Methodology

**FIGURE 1:**
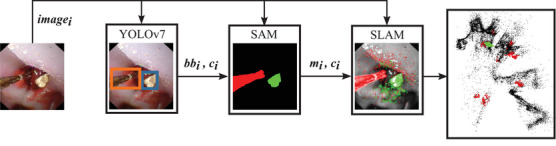
Structure of the proposed SLAM system: The same image $image_{i}$ is fed to the YOLOv7 network, to the SAM network, and also to the SLAM system. First, YOLOv7 estimates the bounding boxes $bb_{i}$ and the classes $c_{i}$ for both object classes. Then, SAM segments the content of the bounding boxes and sends the precise objects' masks ($m_{i}$) to the SLAM system. And last, the SLAM system estimates the 3D point map and the pose of the camera (green rectangle) in real time, using the masks $m_{i}$ to mark the kidney stones in the map (red dots).

As discussed in Section 2.1, the work of Oliva Maza et al. [8] assumed a static environment, but fURS involves dynamic elements such as surgical tools and kidney stones. To handle keypoints detected on these dynamics objects differently, it is necessary to precisely locate them in the images. To achieve this, we propose two approaches: using YOLO11m‐seg (YOLO‐seg), or integrating the YOLOv7‐E6E object detector with the SAM framework (YOLO+SAM). For YOLO+SAM, first YOLOv7‐E6E is used to rapidly detect these objects and generate bounding boxes, while second, SAM performs pixel‐wise segmentation within these bounding boxes. The segmentation masks generated by either of the proposed approaches are then incorporated into the SLAM system from Oliva Maza et al. [8] (see Figure 1), improving endoscope pose estimation and thereby facilitating the surgeon's navigation during the procedure.

For the YOLO‐seg approach, it is necessary to fine‐tune YOLO11m‐seg using pixel‐wise ground truth masks, whereas for the YOLO+SAM approach, only the YOLOv7‐E6E object detector needs to be fine‐tuned using bounding box annotations. To fine‐tune both networks for the detection and classification of tools and kidney stones, we recorded and annotated two datasets: dataset_phantom and dataset_real (Table 1). Note that the segmentation network SAM is agnostic to the object class and must not be retrained.

**TABLE 1:** Number of images in the training and test set of the recorded datasets.

Name	Training set	Test set
Dataset_phantom	500	161
Dataset_real	34	19

**FIGURE 2:**
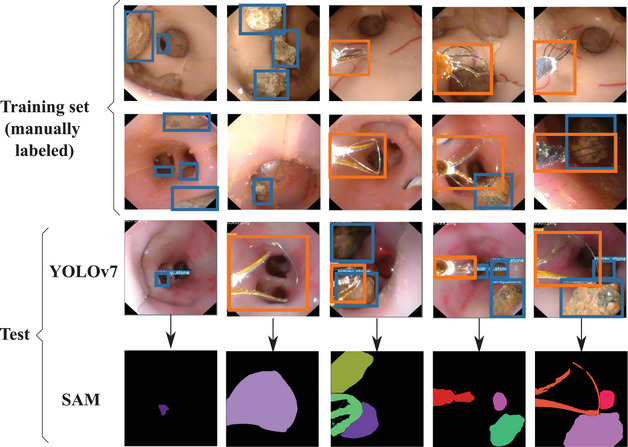
Dataset_phantom images with bounding boxes. The first two rows belong to the labelled training set, while the third row belongs to the test set. The output on the test set is separated in its detection output using YOLOv7 (third row) and the segmentation output using both the bounding boxes of YOLOv7 and the SAM network (fourth row).

Dataset_phantom was acquired using a commercially available flexible ureteroscope Olympus URF‐V^1^, two different types of stones (expelled kidney stones and fake kidney stones), 3 different types of baskets with different shapes and three different kidney phantoms^2^, ^3^. Out of three phantoms, two have been used for training and one for testing (Figure 2).

**FIGURE 3:**
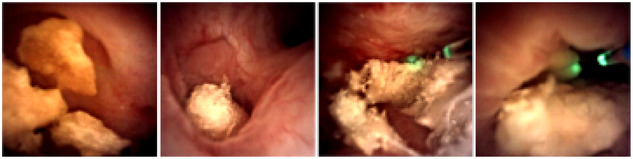
Images belonging to a real kidney stone removal procedure (dataset_real). They contain kidney stones and fibre lasers.

Dataset_real (Figure 3) is a smaller dataset recorded during different kidney stone removal procedures at the University Hospital Freiburg, using the LithoVue Single‐Use Digital Flexible Ureteroscope^4^. In this dataset, baskets and laser fibres are present as well as stones of different types and compositions.

**FIGURE 4:**
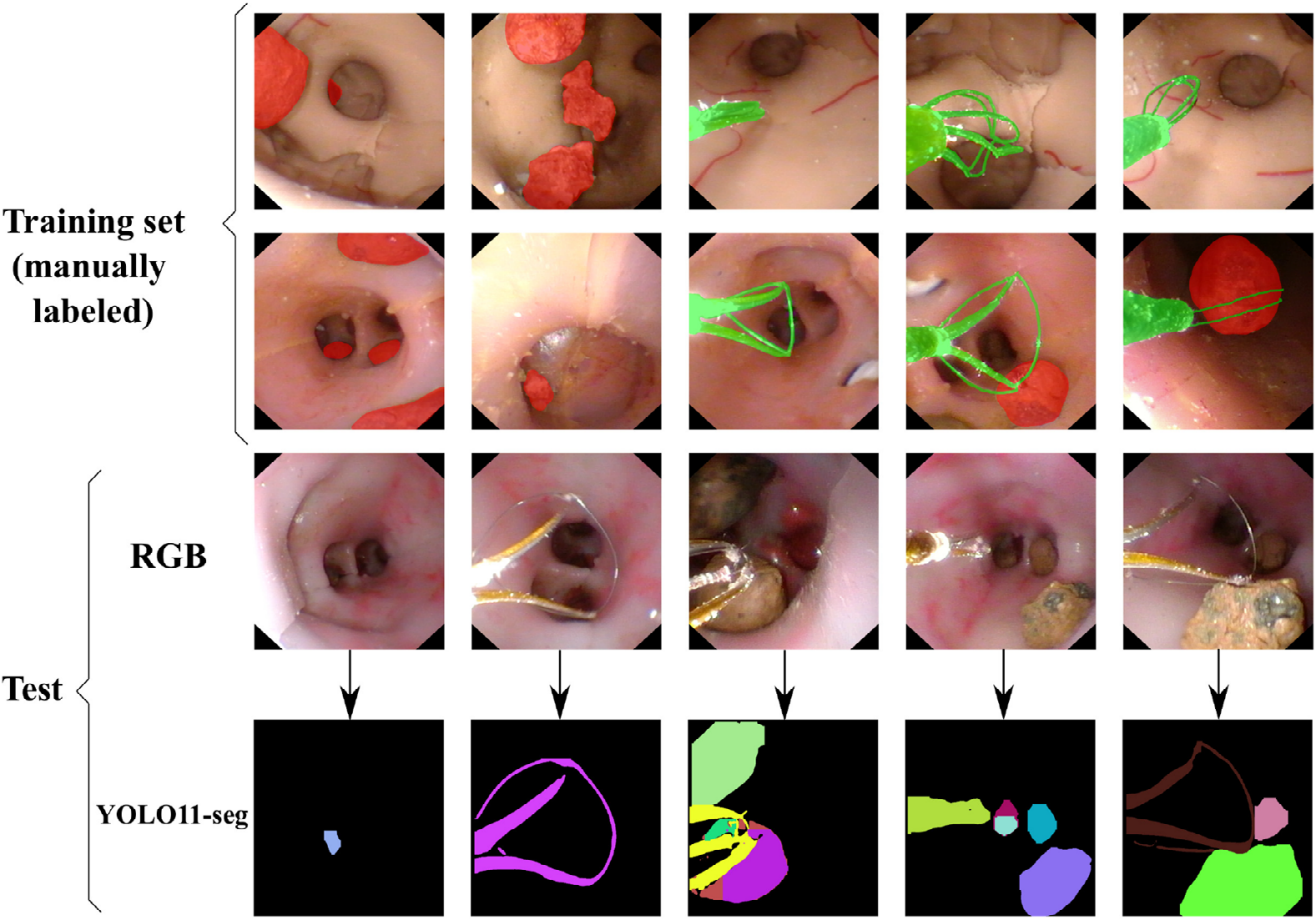
Dataset_phantom images with segmentation masks. The first two rows belong to the labelled training set, while the third row belongs to the test set. The estimated masks by YOLO11‐seg on the test can be seen in the last row.

Both datasets were manually annotated with bounding boxes (using LabelImg^5^ ‐ Figure 2) and segmentation masks (using SAMAT – SAM Annotation Tool^6^, without leveraging SAM's automatic segmentation capabilities ‐ Figure 4). The segmentation masks were subsequently converted into open polygons to enable fine‐tuning of YOLO11m‐seg.

The models were trained and evaluated using different combinations of both datasets. Details of the training and evaluation procedures are provided in the following section.

The next step is to leverage the predicted mask in the SLAM system. When an image is received, A‐KAZE features are extracted to estimate the camera pose by matching them with the projected map points. If a new area is explored, a new keyframe is created and map points are added. Since ORB‐SLAM3 assumes a static environment, we use the estimated segmentation mask to identify dynamic objects like tools, which keypoints are then ignored during pose estimation. Kidney stones are treated differently: keypoints detected on a kidney stone are labelled as kidney stone map points and displayed in a different colour on the map to assist with navigation and ensure complete removal of all stones. When a stone is removed, the system detects its absence (i.e., the kidney stone map points projected into the camera are no longer match with the keypoints extracted in the image) and updates the map by removing the kidney stone map points, ensuring unbiased camera pose estimation.

To further enhance robustness, we add a verification step to prevent incorrect kidney stone map points due to inaccuracies in the classification or the segmentation mask. For a map point to be marked as a kidney stone, it must be consistently projected onto a stone mask for five consecutive frames, a number that is empirically tuned for optimal performance. The same process applies when removing stone map points.

Our objective is to evaluate whether the additional complexity of mask‐level labelling provides a significant advantage, or if simpler bounding‐box‐based labelling already suffices to enhance SLAM accuracy.

## 4 Experiment Results and Discussion

This section evaluates the proposed approach, focusing on the classification and segmentation accuracy of YOLO+SAM and YOLO‐seg, as well as the performance of the SLAM system when using predicted segmentation masks in comparison to the original system. All experiments were performed with a 13th Gen Intel Core i9‐13900K CPU (24 cores), 64GB RAM, and a Nvidia GeForce RTX 4090 24GB GPU.

### 4.1 Segmentation of Kidney Stones and Tools

The first part of the evaluation focuses on assessing the accuracy of classification and segmentation masks across different models. Specifically, we fine tuned four models: **YOLO+SAM_T1**, a YOLOv7‐E6E model fine tuned exclusively on images from various kidney phantoms (training set of dataset_phantom); **YOLO+SAM_T2**, a YOLOv7‐E6E model fine tuned on both kidney phantom images and real kidney images (training sets of both datasets); **YOLO‐seg_T1**, a YOLO11m‐seg model fine tuned on the same data as YOLO+SAM_T1; and **YOLO‐seg_T2**, a YOLO11m‐seg model fine tuned on the same data as YOLO+SAM_T2. The objective is to evaluate the models' generalization ability (from phantom data to real data, comparison between T1 and T2 models) and to compare the performance of YOLO+SAM with YOLO‐seg.

The results of this evaluation are presented in two parts: instance segmentation and semantic segmentation. Instance segmentation assesses how accurately the model detects and classifies individual objects in the image, whereas semantic segmentation measures the pixel‐wise accuracy of the predicted masks for each class. Both evaluations are performed on the test set of dataset_real.

The results of the instance segmentation evaluation are reported in Table 2. For this analysis, we computed the precision and recall of each model per class, considering an object as correctly detected if the intersection over union (IoU) between the predicted mask and the ground truth mask exceeded 0.5. From the results in Table 2, it can be observed that YOLO+SAM achieves higher precision but finds correctly fewer objects (lower recall) compared to YOLO‐seg. When assessing the generalization capabilities of both approaches, the T2 models demonstrate improvements in both precision and recall relative to their respective T1 counterparts. The most notable gains are observed in the tool class, likely due to the fibre laser's consistent and simple appearance. For the kidney stone class, although precision and recall also improve, detection remains challenging because of variations in stone shape and composition, as well as reduced visibility caused by dusting. Addressing these challenges would require a larger set of real training images.

**TABLE 2:** Instance segmentation evaluation of the fine tuned models on kidney stone removal procedures. Note that a baseline using the standard YOLOv7‐E6E or YOLO11m‐seg is meaningless as the network lacks of trained “kidney stone” and “tool” classes.

		YOLO+SAM_T1	YOLO+SAM_T2	YOLO‐seg_T1	YOLO‐seg_T2
Precision	Kidney stone	0.7	0.9	0.45	0.59
	Tool	1.0	0.86	0.29	1.0
Recall	Kidney stone	0.58	0.69	0.91	1.0
	Tool	0.06	0.38	0.14	0.76

The results of the semantic segmentation evaluation are presented in Table 3. For the kidney stone class, YOLO+SAM produces more precise masks, likely because of the similarity between kidney stones and real stones, an object category already encountered by SAM during training. In contrast, tools are often segmented incorrectly by SAM, probably due to the limited representation of such objects in its training data while YOLO‐seg is able to accurately estimate the tool mask after fine‐tuning in real data (YOLO‐seg_T2). Regarding generalization, the same trend observed in the instance segmentation evaluation is observed here: adding a small number of real images to the training set leads to clear improvements, particularly for the *tool* class.

**TABLE 3:** Semantic segmentation evaluation of the fine tuned models on kidney stone removal procedures. Note that a baseline using the standard YOLOv7‐E6E or YOLO11m‐seg is meaningless as the network lacks of trained “kidney stone” and “tool” classes.

	Object	YOLO+SAM_T1	YOLO+SAM_T2	YOLO‐seg_T1	YOLO‐seg_T2
IoU	Kidney stone	0.68	0.71	0.48	0.65
	Tool	0.15	0.36	0.08	0.69
Precision	Kidney stone	0.69	0.74	0.5	0.67
	Tool	0.16	0.44	0.1	0.71
Recall	Kidney stone	0.69	0.75	0.68	0.76
	Tool	0.15	0.38	0.09	0.76
Dice	Kidney stone	0.37	0.42	0.39	0.6
	Tool	0.05	0.3	0.09	0.63

Overall, while YOLO+SAM enables a faster and simpler labelling process and provides more precise segmentation masks for kidney stones, YOLO‐seg achieves superior overall performance. In addition, its inference is more efficient, operating at 55 Hz compared to 10 Hz for YOLO+SAM. Therefore, YOLO‐seg represents the more suitable choice for real‐time clinical deployment.

### 4.2 Performance of the SLAM System

This subsection evaluates the accuracy of the vSLAM system using a dataset recorded with the setup detailed in Section  3. Ground truth data was obtained by electromagnetically tracking the ureteroscope in 6 degrees of freedom with the NDI Aurora system. The 6DoF sensor used has a position accuracy error of 0.8 mm and an orientation accuracy error of 0.7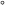. During recording, the endoscope was held by a robotic arm [6], simplifying tool insertion and operation.

We recorded 7 different sequences: *Sequence 1* involves normal kidney exploration without tool insertion; *Sequences 2–5* are normal kidney explorations with a tool inserted at the end of the sequence to grab a stone; *Sequences 6 and 7* have a tool introduced at the beginning and throughout most of the sequence.

The absolute trajectory error (ATE), $\varepsilon_{\text{traj}}=\sqrt{\sum_{t=0}^{N}{\left(\frac{1}{N}x_{t}-{\tilde{x}}_{t}\right)}^{2}}$, with N being the total number of frames, is used to evaluate the trajectory. It calculates the RMSE of the global positions $x_{t}$ of the estimated trajectory compared to the ground truth ${\tilde{x}}_{t}$, after both trajectories are temporally and geometrically aligned following the proposed method by Sturm et al. [30].

Table 4 presents the mean and the standard deviation of $\varepsilon_{\text{traj}}$ over 5 executions to compensate for the randomness introduced by RANSAC used in the SLAM algorithm. The results show that, overall, using the segmentation mask to exclude keypoints detected on the tools enhances SLAM accuracy and robustness. This improvement is particularly noticeable when the tool is introduced at the beginning of the trajectory and remains present for most of the sequence (*Sequences 6 and 7*). In these cases, the SLAM system of Oliva Maza et al. [8] relies on dynamic keypoints from the tool for the map initialization, resulting in incorrect endoscope pose estimations. In contrast, our approach ignores these keypoints, leading to more accurate endoscope trajectory estimates with low error.

**TABLE 4:** Mean (standard deviation) of the absolute trajectory error $\varepsilon_{\text{traj}}$ over 5 runs of the SLAM system in mm.

Sequence	Oliva Maza et al. [8]	YOLO+SAM	Difference (%)	YOLO‐seg	Difference (%)
*Sequence 1*	2.87 mm (±0.25)	2.72 mm (±0.23)	5.24%	2.42 mm (±0.21)	15.72%
*Sequence 2*	1.63 mm (±0.52)	1.23 mm (±0.10)	30.74%	0.9 mm (±0.17)	44.91%
*Sequence 3*	5.35 mm (±6.78)	3.47 mm (±0.84)	35.08%	1.69 mm (±0.1)	68.41%
*Sequence 4*	1.09 mm (±0.13)	1.14 mm (±0.05)	−2.95%	0.81 mm (±0.03)	25.78%
*Sequence 5*	2.89 mm (±0.76)	1.73 mm (±1.06)	40.29%	1.19 mm (±0.04)	58.79%
*Sequence 6*	14.25 mm (±9.24)	4.54 mm (±2.19)	68.75%	2.72 mm (±0.56)	80.89%
*Sequence 7*	3.73 mm (±2.89)	1.05 mm (±0.03)	71.77%	1.01 mm (±0.03)	72.95%

Replacing YOLO+SAM with YOLO‐seg yields further improvements in the SLAM system, primarily due to the increased precision of the tool masks. This enhanced accuracy is illustrated by the comparison between the YOLO+SAM masks shown in Figure 2 and the YOLO‐seg masks presented in Figure 4.

As mentioned in Section 1, we provide a GUI with an augmented navigation map to help the surgeon locate the kidney stones, as shown in Figure 1. Specifically, the visual navigation keypoints corresponding to the kidney stones are used for this visualization, reassuring the surgeon that no stone is left behind.

While this section mainly addresses the advantages of our approach, it is worth mentioning that its main drawback is the framerate reduction from 55 to 28Hz in the case of YOLO‐seg and 9Hz in the case of YOLO+SAM, owing to the calculation of the segmentation masks. The framerate is determined by the time it takes to process one frame, that is, feature extraction and pose estimation for the system from Oliva Maza et al. [8] and estimation of bounding box, mask prediction, feature extraction and pose estimation for the proposed algorithm.

## 5 Conclusions

In this paper we propose an extension of Oliva Maza et al. [8] in which we estimate kidney stone and surgical tools masks by using the combination of the YOLOv7‐E6E object detector and the SAM segmentation networks or by using YOLO11m‐seg. With this, we aim to improve the SLAM system by relaxing the assumption that the environment is static and dealing with dynamic objects. We propose to ignore the visual features detected on surgical tools and to still use the features detected on kidney stones yet to mark them in the map, facilitating the navigation for the surgeon and the tracking of the stones. Besides, we also mark the kidney stone map points as dynamic, to inform the SLAM system that those map points might disappear, automatically updating the map when a kidney stone is removed.

To estimate the segmentation masks, we evaluated the combination of the YOLOv7‐E6E object detector with the SAM segmentation network against YOLO11m‐seg. The YOLOv7‐E6E and SAM combination was selected for its accuracy and speed, as well as the convenience of labelling newly acquired datasets using bounding boxes. YOLO11m‐seg was chosen due to its high accuracy and fast inference time. To fine tune YOLOv7‐E6E and YOLO11m‐seg on the new object classes “tool” and “kidney stone”, we have recorded and annotated two datasets: dataset_phantom using three kidney phantoms (two for gathering training data, one for testing), two types of kidney stones, and three types of surgical baskets and dataset_real consisting of images of real kidney stone removal procedures. The use of the dataset_phantom for fine tuning the models YOLO+SAM_T1 and YOLO‐seg_T1 show lower generalization capabilities to real kidney images (dataset_real). To improve the result on real kidney images, we successfully fine tuned two new models, YOLO+SAM_T2 and YOLO‐seg_T2, with the images of both datasets. YOLO‐seg demonstrated higher precision and recall, more accurate pixel‐wise mask estimations for all objects, and a higher frame rate. The approach YOLO+SAM can be used to facilitate the data annotation needed to fine tune YOLO11m‐seg after reviewing and refining the estimated YOLO+SAM masks.

Finally, we recorded a ground‐truth dataset to show that, indeed, by blocking the surgical tool features, the average improvement in the accuracy of the SLAM system over the 7 sequences is 35.4% when using YOLO+SAM and 52.49% when using YOLO‐seg. The visualization of the position of the kidney stones on the map has the potential to facilitate navigation tasks, which will be investigated within user studies in the future.

Kidney stone segmentation is just the prior step for other related tasks like estimating its size, estimating the path from the camera to the kidney stone, and also to keep improving the SLAM system by using those keypoints.

Future work will include recording a bigger dataset of more kidney stone removal procedure to improve the detection of stones and tools. We also plan to investigate how the reduction in frame rate impacts real‐time clinical application and work on improving processing speed to achieve real‐time performance.

## Author Contributions

**Laura Oliva Maza**: conceptualization, data curation, investigation, methodology, resources, software, validation, writing – original draft, writing – review and editing. **Florian Steidle**: supervision, writing – review and editing. **Julian Klodmann**: supervision, writing – review and editing. **Klaus H. Strobl**: supervision, writing – review and editing. **Arkadiusz Miernik**: supervision, writing – review and editing. **Rudolph Triebel**: supervision.

## Funding

The authors have nothing to report.

## Conflicts of Interest

The authors declare no conflicts of interest.

## Data Availability

Research data are not shared.

## Endnotes

1 Olympus Deutschland GmbH, Hamburg, Germany

2 SAMED GmbH, Dresden, Germany

3 Max Planck Institute, Munich, Germany

4 Boston Scientific, Marlborough, Massachusetts, U.S

5
https://github.com/HumanSignal/labelImg

6
https://github.com/Divelix/samat
